# The Treatment of Ovarian Cysts by Drainage

**Published:** 1876-09

**Authors:** 


THE TREATMENT OF OVARIAN CYSTS BY DRAINAGE.—In a paper read before the Societe de Chirurgie on March 29th, 1876, M. Delore advocates the treatment of ovarian cysts by abdomino-vaginal drainage, in cases in which there is reason to believe that there is a very short pedicle, or none at all, and when a part of the cyst is prominent in the vagina, especially when the existence of pelvic adhesions is probable. The presence of solid masses in the tumor would be a contra-indication to this mode of treatment. The dangers to be avoided are septic inflammation of the cyst and peritonitis. The former is avoided by making an opening into the posterior cul-de-sac, so as to allow a free drainage, and the washing out the cyst by disinfectants. Peritonitis is prevented by determining adhesion by means of caustics, applied both to the abdominal wall and also to the vaginal cul-de-sac. M. Delore records a case in which this treatment was successfully carried out. The patient, aged forty, had an ovarian tumor for three years. By simple tapping, twelve litres of clear fluid were first evacuated, and M. Delore then satisfied himself by means of the blunt end of the canula, that the cyst was in immediate contact with the vaginal cul-de-sac, and that no small cysts or solid matter intervened. The cyst filled again in two months, and the drainage was then established. From ten to thirty litres of water were injected into the cyst every day. At the end of two months the drainage tube was removed and the vaginal opening closed. The abdominal opening also closed, but afterwards opened again, and allowed the escape of a little pus. It closed finally after the injection of a concentrated solution of nitrate of silver. The patient has remained well for two years. *Obstetrical Journal of Great Britain.*

